# An Improved Recognition Approach for Noisy Multispectral Palmprint by Robust L2 Sparse Representation with a Tensor-Based Extreme Learning Machine

**DOI:** 10.3390/s19020235

**Published:** 2019-01-09

**Authors:** Dongxu Cheng, Xinman Zhang, Xuebin Xu

**Affiliations:** 1 School of Electronics and Information Engineering, MOE Key Lab for Intelligent Networks and Network Security, Xi’an Jiaotong University, Xi’an 710049, China; zhangxinman@mail.xjtu.edu.cn; 2 Guangdong Xi’an Jiaotong University Academy, No. 3, Shuxiangdong Road, Daliang, Foshan 528000, China; ccp9999@126.com

**Keywords:** multispectral palmprint recognition, robust L2 sparse representation, WSCCI, adaptive weighted fusion, tensor based ELM

## Abstract

For the past decades, recognition technologies of multispectral palmprint have attracted more and more attention due to their abundant spatial and spectral characteristics compared with the single spectral case. Enlightened by this, an innovative robust L2 sparse representation with tensor-based extreme learning machine (RL2SR-TELM) algorithm is put forward by using an adaptive image level fusion strategy to accomplish the multispectral palmprint recognition. Firstly, we construct a robust L2 sparse representation (RL2SR) optimization model to calculate the linear representation coefficients. To suppress the affection caused by noise contamination, we introduce a logistic function into RL2SR model to evaluate the representation residual. Secondly, we propose a novel weighted sparse and collaborative concentration index (WSCCI) to calculate the fusion weight adaptively. Finally, we put forward a TELM approach to carry out the classification task. It can deal with the high dimension data directly and reserve the image spatial information well. Extensive experiments are implemented on the benchmark multispectral palmprint database provided by PolyU. The experiment results validate that our RL2SR-TELM algorithm overmatches a number of state-of-the-art multispectral palmprint recognition algorithms both when the images are noise-free and contaminated by different noises.

## 1. Introduction

Palmprint recognition technologies have become a novel biometric approach and have attracted increasingly attention in recent years. In comparison with some other biological features (i.e., the iris and fingerprints, etc.), palmprints have a larger collection area with more abundant information. Besides, palmprints possess the characteristics of uniqueness, stability, scalability and non-contact acquisition, etc. As a consequence, they have strong anti-noise capability and efficient discrimination performance.

The current palmprint recognition algorithms can be mainly categorized into various sorts, such as subspace-based methods, feature-based methods and sparse representation-based classification (SRC) methods, etc. The subspace-based methods [1,2,3,4,5,6,7,8,9,10] adopt dimension reduction theory to accomplish the feature space transformation. This can reduce the data complexity and efficiently improve the discrimination of image characteristics. The conventional subspace transformation methods mainly includes the principal component analysis (PCA) [1], linear discriminant analysis (LDA) [4] and independent component analysis (ICA) [7], etc. However, due to their sensitivity to lighting, noise and other contaminations, the conventional linear discriminant methods already don’t meet the requirements of actual palmprint recognition problems. To address these issues, a nonlinear spatial structure transformation technique, namely the kernel PCA method [9,10], was introduced into the palmprint recognition field. In addition, lots of feature-based methods were presented to implement palmprint recognition tasks. For instance, the coding-based method [11,12,13,14,15,16,17] has been extensively researched in the past decades. In these studies, the palmprint features were extracted by using the coding of some filtering results. The common coding methods include binarized statistical image features (BSIF) [13], double-orientation code (DOC) [14] and block dominate orientation code [17], etc. Minaee et al. [18] proposed a palmprint recognition algorithm by using the deep scattering network which achieved fine recognition performance. Some other feature- based methods [19,20,21,22,23,24] mainly take advantage of the statistic characteristics, such as mean, variance, and covariance and so on, to implement palmprint recognition. In recent years, the linear representation methods based on sparse theory [25] were proposed and popularly applied to the palmprint recognition problem [26,27,28,29,30,31]. These methods consider a testing sample as a linear representation of the training set. That is, a given testing sample was anticipated to be approximately expressed by the training samples lied in a unitary class. This can be effectively accomplished by imposing the sparseness constraint on the approximate representation with the training samples.

For the sake of higher recognition accuracy, some multispectral palmprint recognition methods [32,33,34,35,36,37,38,39,40,41,42,43,44] have been studied. Because the collected images under different spectra contain more plentiful feature information, the recognition rate can be effectively improved. In these studies, different fusion strategies were utilized to increase the recognition accuracy. The conventional multispectral palmprint recognition methods can be mainly categorized into image level fusion strategies and matching score level fusion strategies. The basic idea of image level fusion is to decompose the images under different spectra at the start, then integrate these separated decompositions for a compound approximation and reconstruct the fusion image through the inverse transformation to implement the recognition task. Based on this, Han et al. [32] used the discrete wavelet transform (DWT) method to decompose palmprint images acquired under different spectra, and then reconstructed the fused palmprint image to accomplish the multispectral palmprint recognition. Xu et al. [37] introduced the quaternion matrix to represent the palmprint images under different spectra, and then extended PCA and DWT into the quaternion domain to implement feature extraction. Finally, the Euclidean distance was used to perform the recognition task. Gumaei et al. [38] employed an autoencoder with the regularized extreme learning machine (AE-RELM) to accomplish the multispectral palmprint recognition and effectively improve the accuracy. Xu et al. [39] presented a novel multispectral palmprint recognition algorithm. They used the digital shearlet transform (DST) to implement the image fusion and proposed a multiclass projection ELM (MPELM) to accomplish the classification task. For the score level fusion method, the matching scores are obtained separately by a comparator for different spectral bands firstly, then the obtained matching scores are fused by utilizing some rules and accomplish the classification based on the fusion score. Zhang et al. [41] presented a novel algorithm named line orientation-based coding (LOC) to extract the featurew of the palmprint images with different spectrq, and then carried out the recognition task with a matching level fusion rule. Minaee et al. [42] used the co-occurrence matrix to extract the texture features, then employed the minimum distance classifier (MDC) and weighted majority voting system (WMV) to accomplish the multispectral palmprint recognition. Minaee et al. [43] presented a set of wavelet-DCT features for multispectral palmprint recognition. Although many achievements have been made in the study of multispectral palmprint recognition, there are still many open questions that need to be further studied. For example, how to increase the recognition accuracy when the collected images are contaminated by different noises.

Inspired by the these studes, in this article, we present a novel robust L2 sparse representation with a tensor-based extreme learning machine (RL2SR-TELM) algorithm by using an adaptive image level fusion strategy to accomplish the multispectral palmprint recognition. The key contributions of our algorithm can be summarized as follows: Firstly, a robust L2 norm-based sparse representation model is constructed to calculate the linear representation coefficients. It overcomes the defects of high computational complexity of the L1 norm regularization and the lack of robustness to noise contamination. Secondly, an adaptive weighted method is presented to accomplish the fusion of multispectral palmprint images at the image level. In this method, a weighted sparse and collaborative concentration index (WSCCI) is proposed that can quantify the multispectral palmprint image discrimination efficiently. By using the robust sparse coefficients and WSCCI, an adaptive weighted fusion strategy is proposed to reconstruct the fused palmprint image. Finally, aiming at the high order signal classification problem, we extend the conventional ELM [45] into the tensor space, then put forward a novel TELM method. It inherits the advantages of the conventional ELM (i.e., excellent learning speed and generalization performance) which achieves an outstanding recognition efficiency.

The rest of this paper is organized as follows: in Section 2, we introduce the principle of multispectral palmprint acquisition device. Then we discuss our proposed RL2SR-TELM algorithm in Section 3. In Section 4, simulation experiments and the result analysis of our proposed algorithm are illustrated in detail. Section 5 concludes this paper.

## 2. Acquisition Device of Multispectral Palmprint Images

**Figure 1:**
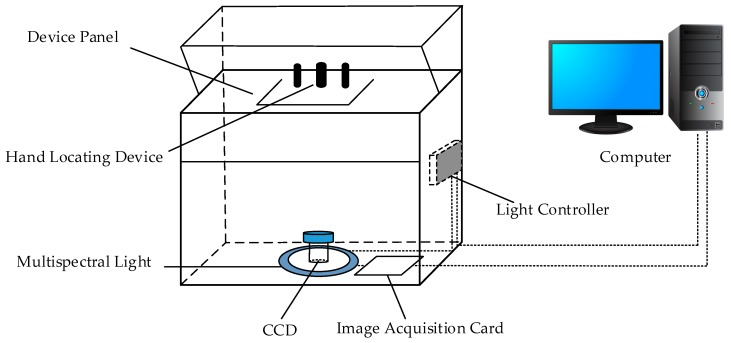
Principle of the multispectral palmprint acquisition device.

**Figure 2:**
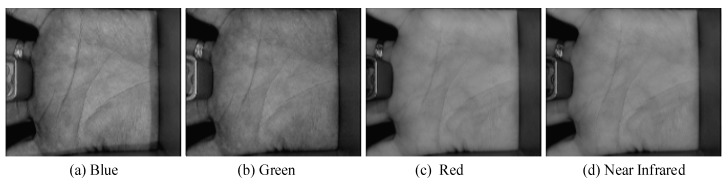
Palmprint images acquired with different spectrums.

The Biometrics Research Centre (BRC) of Hong Kong Polytechnic University (PolyU) has developed an acquisition device [46] for multispectral palmprints. It can collect the palmprint images using the Blue, Green, Red and Near Infrared (NIR) spectra, respectively. Figure 1 illustrates the principle of the acquisition device. It mainly includes a multispectral light source module, a light source control module, a CCD imaging sensor, an image acquisition module (A/D conversion module) and an image display module, etc. The multispectral light source module locates at the bottom of the device and consists of four monochromatic light sources. The light controller module controls the multispectral light and enables CCD imaging module to acquire palmprint images under different spectrums. The image acquisition module captures the multispectral palmprint images and converts analog image into a digital one by an A/D conversion. Figure 2 shows the acquired palmprint images with different spectrums.

## 3. Proposed Algorithm

**Figure 3:**
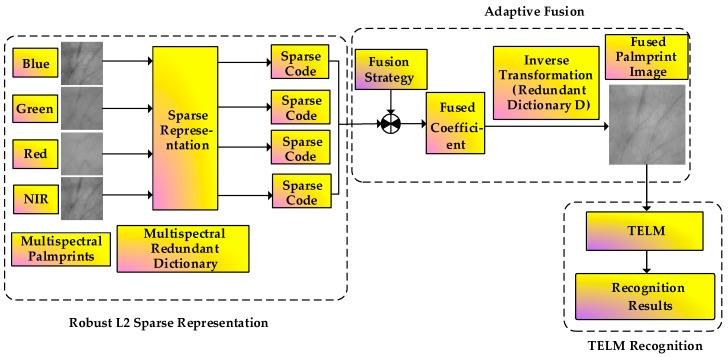
Flowchart of the proposed RL2SR-TELM algorithm.

Figure 3 illustrates the flowchart of the presented RL2SR-TELM algorithm. It can be mainly separated into the following steps: Firstly, the acquired multispectral palmprint image is preprocessed to obtain the region of interest (ROI) of the image. Then, we calculate the sparse representation coefficients of sample images under different spectra by utilizing the proposed robust L2 sparse representation method. After that, an adaptive weighted fusion strategy is presented to obtain the fused images. Finally, by integrating the tensor theory with ELM, we propose a TELM method to complete the recognition task.

### 3.1. Robust L2 Sparse Representation Method

#### 3.1.1. SRC Model

The sparse representation idea was introduced into the biometric recognition for the first time in 2009 by Wright et al. Given the training set matrix denoted as $X=[x_{1},x_{2},\ldots ,x_{n}]\in R^{d\times n}$, where $x_{i}$ is a training sample, d and n denote the training sample dimension and number, respectively. For any given testing sample $y\in R^{d}$, we suppose that it can be coded over the training matrix X approximately, then the SRC model can be described as:

(1) $$\alpha =\arg\underset{\alpha }{\min}{\left\|\alpha \right\|}_{0},\;s.t.\;y=X\alpha ,$$

where $\alpha \in R^{n}$ is the representation coefficient, ${\left\|\alpha \right\|}_{0}$ denotes the L0 norm and it counts the nonzero element number of the vector. The objective of SRC is to find as fast as possible as sparse coefficient α which can represent the testing sample over the training set. Model (1) is a NP hard problem and theoretically intractable. Reference [47] has proved that when the representation coefficient is sparse enough, the L0 norm can be approximately represented by using the L1 norm. On the basis of this theory, Wright et al. proposed the following model:

(2) $$\alpha =\arg\underset{\alpha }{\min}{\left\|\alpha \right\|}_{1},\;s.t.\;y=X\alpha .$$

This is a classical model and it has been extensively used in various areas including the image reconstruction, image de-noising, compressive sensing and machine learning, and so on. Although many scholars have devoted themselves to this algorithm and proposed largely improvements, the drawback of inefficiency is still not completely resolved.

#### 3.1.2. Robust L2 Sparse Representation Method

A SRC model actually supposes that the coding residual obeys a Gaussian or Laplacian probability density function distribution. However, this hypothetical description is not always accurate enough in practice. In addition, the SRC needs to solve the L1 regularization problem and its calculation speed is very slow. To address these drawbacks, many researchers have proposed a lot of improved SRC algorithms. For examples, Yang et al. [48] proposed a novel sparse representation method that solved the sparse representation problem by using the maximum likelihood estimation (MLE) method. It can deal with the occlusion and outliers more robustly. Xu et al. [49] made use of the L2 regularization to acquire the sparse coefficient and proposed a new discriminative sparse representation method (DSRM). Inspired by these ideas, we propose a novel robust L2 regularization based sparse representation method, namely RL2SR.

Suppose that there are s different spectral bands, the class number of each spectral palmprint is C and each class has m training samples. Thus, there are N=mC training samples for each spectrum. Vectorize the training sample into the d- dimensional column vector, then the training sample matrix can be denoted as $X=[X_{1},\ldots ,X_{i},\ldots ,X_{C}]$, where $X_{i}=[x_{m(i-1)+1}^{1},x_{m(i-1)+1}^{2},\ldots ,x_{m(i-1)+1}^{s},\ldots ,x_{mi}^{1},x_{mi}^{2},\ldots ,x_{mi}^{s}]$ is the training sample sub-matrix of the *i*-th class, $x_{mi}^{1},x_{mi}^{2},\ldots ,x_{mi}^{s}$ are the (*m*×*i*)-th training samples under different spectra. Then, given any testing sample $y^{l}$, where l=1, 2, …, s denotes the spectral bands, we can construct the following optimization problem:

(3) $$\arg\underset{A^{l}}{\min}\rho (y^{l}-XA^{l})+\lambda \phi (A^{l}),(l=1,2,\ldots ,s),$$

where λ>0 is a constant namely regularization parameter which can balance the representation residual term and the regularization term. Here, $A^{l}=[A_{1}^{l};A_{2}^{l};\cdots ;A_{C}^{l}]$ is the linear representation coefficient with respect to the testing sample $y^{l}$ over the training set.

For the first term of the optimization function (3), it can be denoted as $\rho (e^{l})=\rho (y^{l}-XA^{l})$, where $\rho (\cdot ):R^{d}\to R$. Then:

(4) $$\rho (e^{l})=\sum_{k=1}^{d}\rho (e_{k}^{l}),$$

where $e_{k}^{l}=\left|y_{k}^{l}-X_{k}A^{l}\right|$ denotes the residual term with respect to the kth element between $y^{l}$ and its approximate linear representation $XA^{l}$. $y_{k}^{l}$ and $X_{k}$ are the *k*-th element of the testing sample and the kth row of the training set matrix, respectively. In general, the residual function ρ(⋅) is designed to minimize the effect generated by the occlusion and outliers. Huber, Cauchy and Welsch functions can be used to express the residual function. In reference [48], Yang et al. utilized the logistic function to describe the residual information and got satisfactory performance. The logistic function can be expressed as follows:

(5) $$\rho (e_{k}^{l})=-\frac{1}{2\mu }(\ln(1+\exp(-\mu {\left(e_{k}^{l}\right)}^{2}+\mu \delta ))-\ln(1+\exp(\mu \delta )))$$

where μ and δ are the positive parameters. The selection of parameters μ and δ will be discussed in Section 4.2. In order to solve question (3), we derivative $\rho (y^{l}-XA^{l})$ with respect to *A^i^*, then we have:

(6) $$\frac{d\rho (y^{l}-XA^{l})}{dA^{l}}=\frac{\sum_{k=1}^{d}d\rho (e_{k}^{l})}{dA^{l}}=\sum_{k=1}^{d}\frac{d\rho (e_{k}^{l})}{de_{k}^{l}}\frac{de_{k}^{l}}{dA^{l}}=\frac{1}{2}\sum_{k=1}^{d}\frac{d\rho (e_{k}^{l})}{de_{k}^{l}}\frac{1}{e_{k}^{l}}\frac{d{\left(e_{k}^{l}\right)}^{2}}{dA^{l}}=\frac{1}{2}\sum_{k=1}^{d}\omega (e_{k}^{l})\frac{d{\left(e_{k}^{l}\right)}^{2}}{dA^{l}}.$$

Furthermore, since $\frac{d}{dA^{l}}({\left\|{\left(W^{l}\right)}^{1/2}e^{l}\right\|}_{2}^{2})=W^{l}\frac{d}{dA^{l}}({\left\|y^{l}-XA^{l}\right\|}_{2}^{2})$, where $W^{l}=diag(\omega (e_{1}^{l}),\omega (e_{2}^{l}),\ldots ,\omega (e_{d}^{l}))$ denotes the residual function, Equation (6) can be regarded as the derivative of $\frac{1}{2}{\left\|{\left(W^{l}\right)}^{1/2}e^{l}\right\|}_{2}^{2}$.

By using Equation (5), the residual function can be calculated as follows:

(7) $$\omega (e_{k}^{l})=\frac{d\rho (e_{k}^{l})}{de_{k}^{l}}\frac{1}{e_{k}^{l}}=\frac{\exp(-\mu {\left(e_{k}^{l}\right)}^{2}+\mu \delta )}{1+\exp(-\mu {\left(e_{k}^{l}\right)}^{2}+\mu \delta )}.$$

For the residual matrix $W^{l}$, the following method is proposed to calculate it:

*Step 1*: Initiate $W^{l,1}=diag(1,1,\ldots ,1)$ and calculate the collaborative code $\gamma^{l}$ of each testing sample by using the collaborative representation model

$$\gamma^{l}=\arg\underset{\gamma^{l}}{\min}{\left\|W^{l}(y^{l}-X\gamma^{l})\right\|}_{2}^{2}+\xi {\left\|\gamma^{l}\right\|}_{2}^{2},(l=1,2,\ldots ,s).$$

*Step 2*: Substitute the collaborative residual $e_{k}^{l}=\left|y_{k}^{l}-X_{k}\gamma^{l}\right|,(k=1,\ldots ,d)$ into Equation (7) and obtain the residual matrix $W^{l}$.

*Step 3*: If $W^{l}$ is not convergent, repeat step 1 and step 2, otherwise output $W^{l}$.

With the residual matrix $W^{l}$ calculated, Equation (3) can be rewritten as follows

(8) $$\arg\underset{A^{l}}{\min}\frac{1}{2}{\left\|{\left(W^{l}\right)}^{1/2}e^{l}\right\|}_{2}^{2}+\lambda \phi (A^{l}),(l=1,2,\ldots ,s).$$

Due to the existence of parameter λ, omit the coefficient in front of the first term and the Equation (8) becomes:

(9) $$\arg\underset{A^{l}}{\min}{\left\|{\left(W^{l}\right)}^{1/2}e^{l}\right\|}_{2}^{2}+\lambda \phi (A^{l}),(l=1,2,\ldots ,s).$$

For the second term $\phi (A^{l})$ of the optimal objective function, SRC [25] adopted the L1 norm to realize the sparseness of linear representation coefficient. In general, an iterative algorithm is employed to solve the L1 norm regularization based sparse representation problem. There are many famous algorithms [50] to implement the iteration, such as L1 regularized least squares (L1LS), homotopy method, augmented Lagrangian method (ALM), orthogonal matching pursuit method (OMP) [51] and fast iterative shrinkage thresholding algorithm (FISTA), etc. However, these methods still suffer from the issue of low efficiency. To address this issue, Zhang et al. [52] introduced the collaborative representation-based classification (CRC) into the method and utilized the L2 regularization to obtain the representation coefficient. Although CRC provided an efficient algorithm, it failed to give full consideration to the sparseness of linear representation. Reference [49] employed L2 regularization to implement the face recognition by utilizing a discriminative sparse representation method. Inspired by this, the L2 regularization item is introduced into our model and a novel RL2SR model is proposed as follows:

(10) $$\arg\underset{A^{l}}{\min}{\left\|{\left(W^{l}\right)}^{1/2}e^{l}\right\|}_{2}^{2}+\lambda \sum_{i=1}^{C}\sum_{j=1}^{C}{\left\|X_{i}A_{i}^{l}+X_{j}A_{j}^{l}\right\|}_{2}^{2},\;(l=1,2,\ldots ,s).$$

Since:

(11) $$\sum_{i=1}^{C}\sum_{j=1}^{C}{\left\|X_{i}A_{i}^{l}+X_{j}A_{j}^{l}\right\|}_{2}^{2}=2\sum_{i=1}^{C}{\left\|X_{i}A_{i}^{l}\right\|}^{2}+2\sum_{i=1}^{C}\sum_{j=1}^{C}\left({\left(X_{i}A_{i}^{l}\right)}^{T}(X_{j}A_{j}^{l})\right),\;(l=1,2,\ldots ,s),$$

Equation (11) can be separated into two parts. Minimizing ${\left(X_{i}A_{i}^{l}\right)}^{T}(X_{j}A_{j}^{l})$ implies that the correlation between the *i*-th class and *j*-th class is also minimal with respect to the linear representation. This makes the linear approximation combination have the best discrimination ability. Thus, the second term of Equation (11) has the capability of decorrelating the linear representation combination with different classes. Correspondingly, minimization of the sum ${\left(X_{i}A_{i}^{l}\right)}^{T}(X_{j}A_{j}^{l})$, instead of any individual terms, can accomplish the decorrelation affection for different classes. In consequence, this approach can discriminate the testing sample to the really nearest class. Minimization of ${\left\|X_{i}A_{i}^{l}\right\|}^{2},(i=1,2,\ldots ,C)$ means that the norm of the linear representation combination with each class is also small. Similar to the presented linear representation approaches, such as SRC and CRC, there is a competitive relationship between different classes of training samples. In other word, the testing sample can be denoted by the weight sum of the training samples from all of the classes. Obviously, that is a linear representation which means every class makes its impact to represent the testing sample. Competition in representation implies that when a class makes an important impact to the linear representation, the remainder classes make considerably less impact.

The objective function shown in Equation (10) can be rewritten as:

(12) $$L(A^{l})=\arg\underset{A^{l}}{\min}{\left\|{\left(W^{l}\right)}^{1/2}(y^{l}-XA^{l})\right\|}_{2}^{2}+\lambda \sum_{i=1}^{C}\sum_{j=1}^{C}{\left\|X_{i}A_{i}^{l}+X_{j}A_{j}^{l}\right\|}_{2}^{2},(l=1,2,\ldots ,s).$$

For the first term of objective function (12), using $\arg\underset{A^{l}}{\min}{\left\|{\left(W^{l}\right)}^{1/2}(y^{l}-XA^{l})\right\|}_{2}^{2}$ instead of $y^{l}=XA^{l}$ implies that $XA^{l}$ is a linear approximation of the test image. That is to say, this model can tolerate considerable noise contamination. In the meantime, the residual function can measure the linear representation residual well and enhance the noise robustness of the proposed model. In order to optimize the presented model, we introduce the following theorem:

**Theorem 1.**  *The proposed RL2SR model (12) is convex and differentiable w.r.t. coefficient*$A^{l}$*, and it has a closed form solution*.

**Proof.**  Firstly, the objective function (12) can be considered as a combination of two L2 regularization terms, i.e., ${\left\|{\left(W^{l}\right)}^{1/2}(y^{l}-XA^{l})\right\|}_{2}^{2}$ and $\sum_{i=1}^{C}\sum_{j=1}^{C}{\left\|X_{i}A_{i}^{l}+X_{j}A_{j}^{l}\right\|}_{2}^{2}$. By adopting the properties of L2 norm, the convexity and derivative of the proposed model (12) can be easily proved.

Secondly, the derivative of function ${\left\|{\left(W^{l}\right)}^{1/2}(y^{l}-XA^{l})\right\|}_{2}^{2}$ can be computed as follows:

$$\frac{d}{dA^{l}}{\left\|{\left(W^{l}\right)}^{1/2}(y^{l}-XA^{l})\right\|}_{2}^{2}=-2X^{T}W^{l}(y^{l}-XA^{l}).$$

On the other hand, for the second term $\sum_{i=1}^{C}\sum_{j=1}^{C}{\left\|X_{i}A_{i}^{l}+X_{j}A_{j}^{l}\right\|}_{2}^{2}$, since it does not contain the coefficient $A^{l}$ explicitly, we could not compute the derivative directly. To address this issue, we compute the partial derivatives of $\sum_{i=1}^{C}\sum_{j=1}^{C}{\left\|X_{i}A_{i}^{l}+X_{j}A_{j}^{l}\right\|}_{2}^{2}$ w.r.t. $A_{k}^{l}(k=1,2,\ldots ,C)$. Denote $\varphi (A^{l})=\sum_{i=1}^{C}\sum_{j=1}^{C}{\left\|X_{i}A_{i}^{l}+X_{j}A_{j}^{l}\right\|}_{2}^{2}$, we have:

$$\begin{array}{ll} \frac{\partial \varphi }{\partial A_{k}^{l}}= & \frac{\partial }{\partial A_{k}^{l}}\left(\sum_{i=1}^{C}\sum_{j=1}^{C}{\left\|X_{i}A_{i}^{l}+X_{j}A_{j}^{l}\right\|}_{2}^{2}\right)\\ & =\frac{\partial }{\partial A_{k}^{l}}\left(\sum_{\begin{array}{l} j=1\\ j\neq k \end{array}}^{C}{\left\|X_{k}A_{k}^{l}+X_{j}A_{j}^{l}\right\|}_{2}^{2}+\sum_{\begin{array}{l} i=1\\ i\neq k \end{array}}^{C}{\left\|X_{i}A_{i}^{l}+X_{k}A_{k}^{l}\right\|}_{2}^{2}+\sum_{\begin{array}{l} i=1\\ i\neq k \end{array}}^{C}\sum_{\begin{array}{l} j=1\\ j\neq k \end{array}}^{C}{\left\|X_{i}A_{i}^{l}+X_{j}A_{j}^{l}\right\|}_{2}^{2}+{\left\|X_{k}A_{k}^{l}+X_{k}A_{k}^{l}\right\|}_{2}^{2}\right)\\ & =\frac{\partial }{\partial A_{k}^{l}}\left(2\sum_{\begin{array}{l} j=1\\ j\neq k \end{array}}^{C}{\left\|X_{k}A_{k}^{l}+X_{j}A_{j}^{l}\right\|}_{2}^{2}+\sum_{\begin{array}{l} i=1\\ i\neq k \end{array}}^{C}\sum_{\begin{array}{l} j=1\\ j\neq k \end{array}}^{C}{\left\|X_{i}A_{i}^{l}+X_{j}A_{j}^{l}\right\|}_{2}^{2}+{\left\|X_{k}A_{k}^{l}+X_{k}A_{k}^{l}\right\|}_{2}^{2}\right)\\ & =4X_{k}^{T}\left(\left(C+1)X_{k}A_{k}^{l}+\sum_{\begin{array}{l} j=1\\ j\neq k \end{array}}^{C}X_{j}A_{j}^{l}\right)\right)\\ & =4X_{k}^{T}\left(CX_{k}A_{k}^{l}+\sum_{j=1}^{C}X_{j}A_{j}^{l})\right)\\ & =4X_{k}^{T}(CX_{k}A_{k}^{l}+XA^{l}) \end{array}$$

Then, we can obtain the derivative as follows:

$$\frac{d\varphi }{dA^{l}}=\left(\begin{array}{c} \frac{\partial \varphi }{\partial A_{1}^{l}}\\ \vdots \\ \frac{\partial \varphi }{\partial A_{C}^{l}} \end{array}\right)=\left(\begin{array}{c} 4X_{1}^{T}(CX_{1}A_{1}^{l}+XA^{l})\\ \vdots \\ 4X_{C}^{T}(CX_{C}A_{C}^{l}+XA^{l}) \end{array}\right)=4C\left(\begin{array}{ccc} X_{1}^{T}X_{1} & \cdots & 0\\ \vdots & \ddots & \vdots \\ 0 & \cdots & X_{C}^{T}X_{C} \end{array}\right)A^{l}+4X^{T}XA^{l}.$$

By denoting:

$$M=\left(\begin{array}{ccc} X_{1}^{T}X_{1} & \cdots & 0\\ \vdots & \ddots & \vdots \\ 0 & \cdots & X_{C}^{T}X_{C} \end{array}\right),$$

we have:

$$\frac{d\varphi }{dA^{l}}=4(CM+X^{T}X)A^{l}.$$

As a consequence, the derivative of objective function (12) with respect to $A^{l}$ is:

$$\frac{dL}{dA^{l}}=-2X^{T}W^{l}(y^{l}-XA^{l})+4\lambda (CMA^{l}+X^{T}XA^{l}),(l=1,2,\ldots ,s).$$

By employing the property of optimal solution, and setting is as zero, the closed solution of objective function (12) is obtained as follows:

(13) $$A^{l}={\left(2\lambda CM+2\lambda X^{T}X+X^{T}W^{l}X\right)}^{-1}X^{T}W^{l}y^{l},(l=1,2,\ldots ,s).$$

The proof of Theorem 1 is thus completed. □

The proposed RL2SR method is summarized in Table 1.

### 3.2. Image Fusion Based on Adaptive Weighted Method

In this section, a weighted sparse and collaborative concentration index is introduced to quantify the discrimination of each spectral testing sample and an adaptive weighted fusion method is proposed to construct the fused palmprint image.

**Definition 1.**  *[25] (sparse concentration index (SCI)) The SCI of a coefficient vector* $\alpha \in R^{n}$ *is defined as:*

(14) $$SCI(\alpha )=\frac{C\cdot \underset{i}{\max}{\left\|\delta_{i}(\alpha )\right\|}_{1}/{\left\|\alpha \right\|}_{1}-1}{C-1},$$

*where* C *is the class number,* $\delta_{i}(\alpha )$ *is an indicator function defined on* $R^{n}$ *which keeps the coefficients affiliated to the* ith *class and sets all the other coefficients to be zero.*

Obviously, SCI(α)=1 implies that the training samples from a unitary class can express the testing sample well. On the contrary, SCI(α)=0 means that all of the training samples have an average impact to represent the testing sample. Therefore, SCI can measure the sparseness of the linear representation coefficient and the discrimination ability of the testing sample efficiently. If SCI(α)=1, the testing sample has the strongest discrimination ability and it can be easily classified into the correct class. If SCI(α)=0, the testing sample has the weakest discrimination ability and we cannot determine the actual class that the testing sample should belong to.

The SCI uses the L1 norm to evaluate the sparseness of the linear representation coefficient and it can’t efficiently evaluate the coefficient obtained by our RL2SR method since the L2 norm regularization is utilized. It considers not only the sparseness, but also the collaborative representation information of the representation coefficient. To address this issue, the definition of SCI is extended and a weighted sparse and collaborative concentration index, namely WSCCI, is proposed to evaluate the representation coefficient obtained by our RL2SR model.

**Definition 2.**  *(weighted sparse and collaborative concentration index (WSCCI)) The WSCCI of a coefficient vector*$\alpha \in R^{n}$*is defined as:*

(15) $$WSCCI(\alpha )=\frac{\mu_{1}\left(C\cdot \underset{i}{\max}{\left\|\delta_{i}(\alpha )\right\|}_{1}/{\left\|\alpha \right\|}_{1}-1\right)+\mu_{2}\left(C\cdot \underset{i}{\max}{\left\|\delta_{i}(\alpha )\right\|}_{2}/{\left\|\alpha \right\|}_{2}-1\right)}{\left(\mu_{1}+\mu_{2})(C-1\right)},$$

*where*C*denotes the class number,*$\mu_{1}$*and*$\mu_{2}$*are nonnegative parameters*.

In WSCCI, the weighted fusion of the sparse and collaborative concentration index defined by the L1 norm and L2 norm is utilized to evaluate the discriminative performance of the given sample. As a consequence, it can be regarded as the weighted sum of SCI and CCI (i.e., collaborative concentration index). From the above analysis, the proposed WSCCI can be utilized to model our adaptive weighted fusion method.

The proposed adaptive weighted image fusion method can be summarized as follows:

(1) For the linear representation coefficients $A^{l},(l=1,2,\ldots ,s)$ obtained by Equation (13), calculate the $WSCCI(A^{l}),(l=1,2,\ldots ,s)$ by using Equation (15).

(2) Normalize $WSCCI(A^{l}),(l=1,2,\ldots ,s)$ by using:

(16) $$\eta^{l}=\frac{WSCCI(A^{l})}{WSCCI(A^{1})+WSCCI(A^{2})+\ldots +WSCCI(A^{s})}=\frac{WSCCI(A^{l})}{\sum_{i=1}^{s}WSCCI(A^{i})},(l=1,2,\ldots ,s).$$

(3) Reconstruct the fused multispectral palmprint image *y* by using:

(17) $$y=X(\eta^{1}A^{1}+\eta^{2}A^{2}+\ldots +\eta^{s}A^{s})=X\sum_{l=1}^{s}\eta^{l}A^{l}.\;$$

With the fused multispectral palmprint image obtained, TELM is proposed to implement the recognition task.

### 3.3. Principle of Tensor Based ELM

ELM can be considered as a generalized single hidden layer feedforward neural network (SLFN). Since ELM randomly chooses the initial values of the hidden nodes and analytically calculates the output weights, the learning speed is extremely fast compared to the conventional supervised learning algorithms (i.e., support vector machine (SVM) [53] and *k*-nearest neighbor (KNN) algorithm, etc.). In addition, its generalization ability is better than many back propagation neural networks algorithms. In consequence, ELM has been extensively studied and widely applied in lots of areas (such as pattern classification, clustering analysis and regression etc.) and plenty of research achievements have been acquired. Inspired by this idea, we present a novel TELM by extend the conventional ELM to the tensor space, and it can regard the image as a tensor to execute the recognition task.

#### 3.3.1. ELM

Given a training set with N different training samples $\left(x_{j},t_{j})\in R^{d}\times R^{m},(j=1,2,\ldots ,N\right)$, where $x_{j}={\left[x_{j1},x_{j2},\ldots ,x_{jd}\right]}^{T}\in R^{d}$ denotes the jth training sample, $t_{j}={\left[t_{j1},t_{j2},\ldots ,t_{jm}\right]}^{T}\in R^{m}$ represents the target of sample $x_{j}$. A classical SLFNs can be theoretically defined by:

(18) $$\sum_{i=1}^{L}\beta_{i}f_{i}(x_{j})=\sum_{i=1}^{L}\beta_{i}f(a_{i}\cdot x_{j}+b_{i})=t_{j},j=1,2,\ldots ,N.$$

In this model, the hidden node number is L and activation function is f(x). $a_{i}={\left[a_{i1},a_{i2},\ldots ,a_{id}\right]}^{T}$ denotes the input weight value which connects the input nodes with the ith hidden node. $\beta_{i}={\left[\beta_{i1},\beta_{i2},\ldots ,\beta_{im}\right]}^{T}$ denotes the output weight value which connects the output nodes with the ith hidden node. $b_{i}$ denotes the bias for the ith hidden node. $a_{i}\cdot x_{j}$ means a dot product between $a_{i}$ and $x_{j}$. The classical SLFNs can approximate the given training samples set with the minimum residual.

Obviously, Equation (18) is a system of linear equations. By introducing the concept of matrix, we can rewrite it as follows:

(19) Hβ=T,

where:

$$H={\left(\begin{array}{ccc} f(a_{1}\cdot x_{1}+b_{1}) & \cdots & f(a_{L}\cdot x_{1}+b_{L})\\ \vdots & \ddots & \vdots \\ f(a_{1}\cdot x_{N}+b_{1}) & \cdots & f(a_{L}\cdot x_{N}+b_{L}) \end{array}\right)}_{N\times L},\beta ={\left(\begin{array}{c} \beta_{1}^{T}\\ \vdots \\ \beta_{L}^{T} \end{array}\right)}_{L\times m},T={\left(\begin{array}{c} t_{1}^{T}\\ \vdots \\ t_{N}^{T} \end{array}\right)}_{N\times m}.$$

**Theorem 2.**  *For a given normative SLFNs which possesses*L*hidden nodes and an activation function*f*, where*f:R→R*is an infinitely differentiable function on the definition interval. Given a training set with*N*different samples*$\left(x_{j},t_{j}\right)$*, where*$x_{j}\in R^{n}$*denotes the sample data and*$t_{j}\in R^{m}$*represents the target of*$x_{j}$*. For any randomly assigned weight*$a_{i}$*and bias*$b_{i}$*, the output matrix*H*of the hidden layer can be obtained by the pseudo-inverse and satisfies*‖Hβ−T‖=0*for probability one with respect to any continuous probability distribution*.

For the proof of the Theorem 2 readers can refer to [45]. Based on this theory, ELM can be descripted as follows: With the initial weight vector and the biases of hidden layer nodes determined by random assignment, we can obtain the output matrix H for the hidden layer based on the input samples. Therefore, we can transform the training procedure of ELM to a classical least squares problem of linear equations, i.e.,

(20) $$\underset{\beta }{\min}{\left\|H\beta -T\right\|}^{2}.$$

We can obtain the least square solution of Equation (20) as follows:

(21) $$\hat{\beta }=H^{+}T.$$

where $H^{+}$ refers to the Moore-Penrose pseudo-inverse for matrix H.

#### 3.3.2. Tensor Based ELM

Although the conventional ELM can deal well with one-dimensional signals, for two-dimensional images, it needs to be vectorized and solved in the one-dimensional space. However, in this transformation it is easy to lose the spatial structure information of the image. In order to solve this problem, we extend the conventional ELM to the tensor space and put forward a novel tensor- based ELM to deal with the high-dimensional signals.

In view of the high-dimensional characteristics of the palmprint image, we regard the fused image as a second-order tensor and classify it by the proposed TELM. In our method, the high order singular value decomposition (HOSVD) algorithm [54] is utilized to decompose the fused palmprint image and construct the input weight values of the TELM model.

Given an M order tensor $F\in R^{I_{1}\times I_{2}\times \cdots \times I_{M}}$ and a matrix $U\in R^{J_{m}\times I_{m}}$, we define $B\in R^{I_{1}\times \cdots \times I_{m-1}\times J_{m}\times I_{m+1}\times \cdots \times I_{M}}$ as the mth modal product of F and U, the elements of B can be calculated by:

(22) $${\left(F\times U_{m}\right)}_{i_{1}\cdots i_{m-1}j_{m}i_{m+1}\cdots i_{M}}=\sum_{i_{n}}f_{i_{1}\cdots i_{m-1}i_{m}i_{m+1}\cdots i_{M}}u_{j_{m}i_{m}}.\;$$

so the *m*-th modal tensor product can be simply denoted by:

(23) $$B=F\times U_{m}.$$

The HOSVD algorithm can be implemented by using the tensor product. Given an M order tensor $F\in R^{I_{1}\times I_{2}\times \cdots \times I_{M}}$, we can use the tensor product to decompose F in the following:

(24) $$F=S\times U^{\left(1\right)}\times U^{\left(2\right)}\times \cdots \times U^{\left(M\right)},$$

where S denotes an M order tensor which is called as a core tensor, $U^{\left(1\right)}\in R^{I_{1}\times I_{1}},\;U^{\left(2\right)}\in R^{I_{2}\times I_{2}},\;\ldots ,$ $U^{\left(M\right)}\in R^{I_{M}\times I_{M}}$ are unitary matrices and each column is corresponding to the orthogonal basis of unfolded matrices $F^{\left(1\right)},\;F^{\left(2\right)},\;\ldots ,\;F^{\left(M\right)}$.

The low rank approximation of tensor F can be calculated by HOSVD, i.e.,

(25) $$F\approx S_{\left(q_{1},\;q_{2},\cdots ,\;q_{M}\right)}\times U_{q_{1}}^{\left(1\right)}\times U_{q_{2}}^{\left(2\right)}\times \cdots \times U_{q_{M}}^{\left(M\right)},$$

where $S\in R^{q_{1}\times q_{2}\times \cdots \times \;q_{M}}$ represents the principal component core tensor, $U_{q_{i}}^{\left(i\right)}$ represents the truncation matrix composed by the first $q_{i}$ columns of $U^{\left(i\right)}$, i=1,2,…,M.

According to the above discussion, we summarize the detailed process of the tensor based ELM as follows: let $G_{i}\in R^{s\times t},(i=1,2,\ldots ,N)$ be the ith fused training palmprint image, $t_{i}\in R^{m},(i=1,2,\ldots ,N)$ be the target of sample $G_{i}$. Denoted the training sample set as $G\in R^{s\times t\times N}$. Then the HOSVD algorithm utilized to decompose $G_{i}\in R^{s\times t},(i=1,2,\ldots ,N)$ can be formulated as:

(26) $$G_{i}\approx S_{\left(L_{1},\;L_{2}\right)}\times U_{L_{1}}\times V_{L_{2}},$$

where $U_{L_{1}}=[u_{1},\;u_{2},\;\ldots ,\;u_{L_{1}}]$ and $V_{L_{2}}=[v_{1},\;v_{2},\;\ldots ,\;v_{L_{2}}]$ represent the truncation matrix with $L_{1}$ and $L_{2}$ columns, respectively. Then TELM can be defined as:

(27) $$\sum_{l_{1}=1}^{L_{1}}\sum_{l_{2}=1}^{L_{2}}g(G_{j}\times u_{l_{1}}\times v_{l_{2}})\beta_{l_{2}+(l_{1}-1)L_{1}}=t_{j}\;,(j=1,\;2,\;\ldots ,\;N),$$

where $L_{1}$ and $L_{2}$ denote the hidden layer node numbers along the tensor directions. In consequence, there are in total $L=L_{1}\times L_{2}$ hidden layer nodes. $u_{l_{1}}$ and $v_{l_{2}}$ denote the input weight vectors of the hidden layer along the tensor directions, respectively. $\beta_{l_{2}+(l_{1}-1)L_{1}}$ denotes the weight value between the output nodes and the $(l_{2}+(l_{1}-1)L_{1})\mathrm{th}$ node in the hidden layer. Similar to ELM algorithm, g(⋅) denotes the activation function. Finally, the output weight β can be obtained from Equation (27) by utilizing the least squares method.

## 4. Experiments

In this section, we evaluate the presented multispectral palmprint recognition algorithm on the benchmark available database offered by PolyU. Extensive experiments are implemented to demonstrate the effectiveness of the presented RL2SR method, adaptive fusion strategy and TELM. In the experiment of this paper, we use the fused palmprint image as the input of TELM classifier. In this section, we accomplish the experiments on a PC equipped with Windows 7, Intel Core i5-2320 CPU (3.0 GHz), and 6 GB RAM, and the algorithm is programmed using MATLAB 2017a.

### 4.1. The PolyU Multispectral Palmprint Database

**Figure 4:**
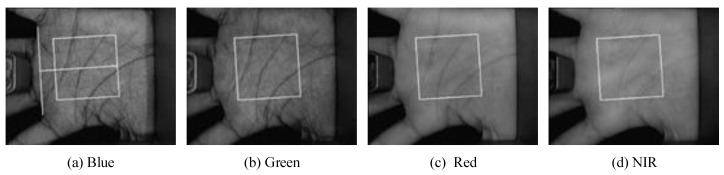
ROI extraction.

The PolyU multispectral palmprint database was taken from 250 persons where the males are 195 and females are 55. The age of volunteers was mainly between 20 and 60 years old. In order to embody the differences of the acquired palmprint and make the palmprint images be various, the palmprint images were acquired in two separate phases. The time interval between the two phases was 5–15 days and each phase lasted about 9 days. In each phase, both hands of the volunteers were acquired six times respectively under the condition of four different spectra: Blue (470 nm), Green (525 nm), Red (660 nm) and NIR (880 nm). For each spectrum, 500 different palmprints were acquired from the 250 volunteers in the two phases. Therefore, the database contains 6000 palmprint images under each spectrum. That is, the multispectral palmprint database contains 6000 × 4 = 24,000 images in total. Reference [46] provided the ROI extraction process from the acquired multispectral palmprint images and established the database namely PolyU multispectral palmprint database (see Figure 4).

**Figure 5:**
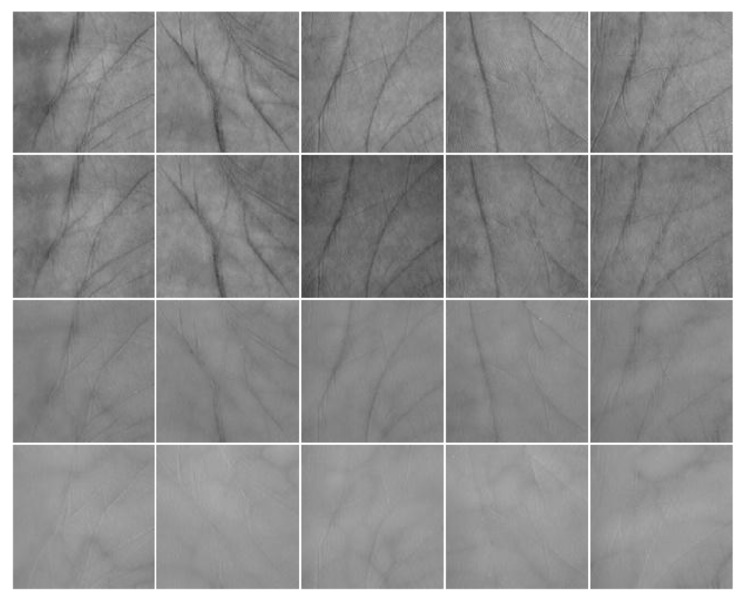
Some multispectral palmprint images of PolyU database.

Figure 5 illustrates some images in the multispectral palmprint database. The images in the rows 1–4 are acquired under the Blue, Green, Red and NIR spectra, respectively. Every column is from the same class. In practice, the acquirement process is easily contaminated by various noises. To simulate this, the white Gaussian noise and salt & pepper noise are added into the images and the recognition experiments are implemented, respectively. 

**Figure 6:**
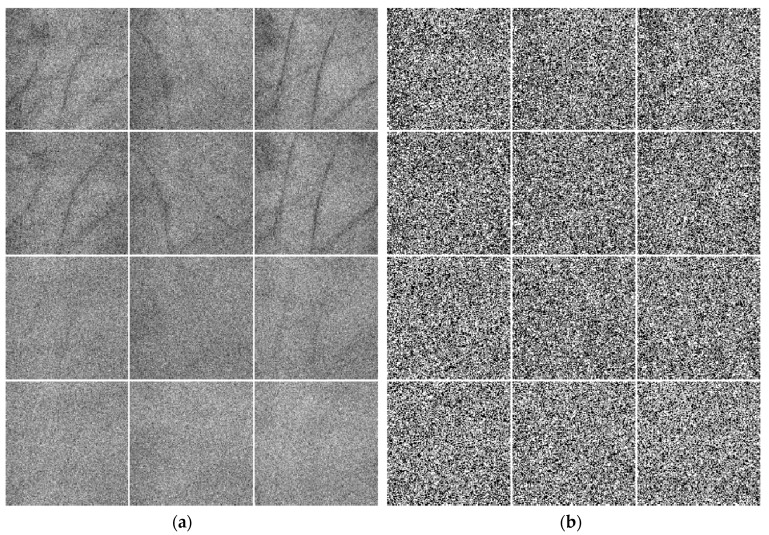
Some multispectral palmprint images of PolyU database contaminated by different noises. (**a**) Palmprint images contaminated by white Gaussian noise. (**b**) Palmprint images contaminated by salt & pepper noise.

Figure 6 displays some multispectral palmprint images contaminated by different noises. Figure 6a shows the images contaminated by white Gaussian noise. Here, the mean is 0 and the standard deviation is 25. Meanwhile, Figure 6b shows the images contaminated by 50% salt & pepper noise. Rows 1–4 of Figure 6 exhibit the noisy palmprint images under the Blue, Green, Red and NIR spectra, respectively.

### 4.2. Parameter Selection

#### 4.2.1. Selection of μ and δ for Residual Function

Now, let’s discuss the selection of parameters μ and δ for the residual function in Equation (7). It can be seen from Equation (7) that $\omega (e_{k}^{l})\to \exp(\mu \delta )/\left(1+\exp(\mu \delta )\right)$ when $e_{k}^{l}\to 0$. Similarly, when $e_{k}^{l}\to \infty$, $\omega (e_{k}^{l})=\exp(\mu \delta )/\left(\exp(\mu {\left(e_{k}^{l}\right)}^{2})+\exp(\mu \delta \right))\to 0$. In order to make ω belong to (0, 1), set the product μδ to be large enough, then $\omega (e_{k}^{l})\approx \exp(\mu \delta )/\exp(\mu \delta )=1$. For simplicity, we denote T=μδ. Since $e^{7}>1000$, in order to meet $\omega (e_{k}^{l})\to 1$ when $e_{k}^{l}\to 0$, set T=μδ>7. From Equation (7), $\omega (e_{k}^{l})=1/2$, when $\delta ={\left(e_{k}^{l}\right)}^{2}$, so the parameter δ determines the boundary point position of the residual function value. That is to say, δ is determined when the weight ω will pass through 0.5. For the sake of enhancing the robustness of the model for the outlier or noise contamination efficiently, a novel method of selecting the parameter δ is presented as follows. Firstly, vectorize the square of the error ${\left(e_{k}^{l}\right)}^{2}$ and denote it as ${\bar{e}}^{l}={\left[{\left(e_{1}^{l}\right)}^{2},{\left(e_{2}^{l}\right)}^{2},\ldots ,{\left(e_{d}^{l}\right)}^{2}\right]}^{T}$, then arrange this vector’s elements in descending order and denote the new vector by ${\tilde{e}}^{l}$. By denoting its maximum element as M and the minimum element as m, set $\tau_{1}=(1-\theta )m+\theta M$, where θ is a constant and θ∈[0.6,0.8]. Since the dimension of ${\tilde{e}}^{l}$ is d, suppose that s is the nearest integer to θd and the sth biggest element of ${\tilde{e}}^{l}$ is selected as $\tau_{2}$. Finally, let $\delta =\left(\tau_{1}+\tau_{2}\right)/2$. Once δ is selected, parameter μ can be calculated by μ=T/δ. In our experiments, select the constant *T* = 8.

#### 4.2.2. Selection of the Hidden Node Numbers Along the Directions of TELM

**Figure 7:**
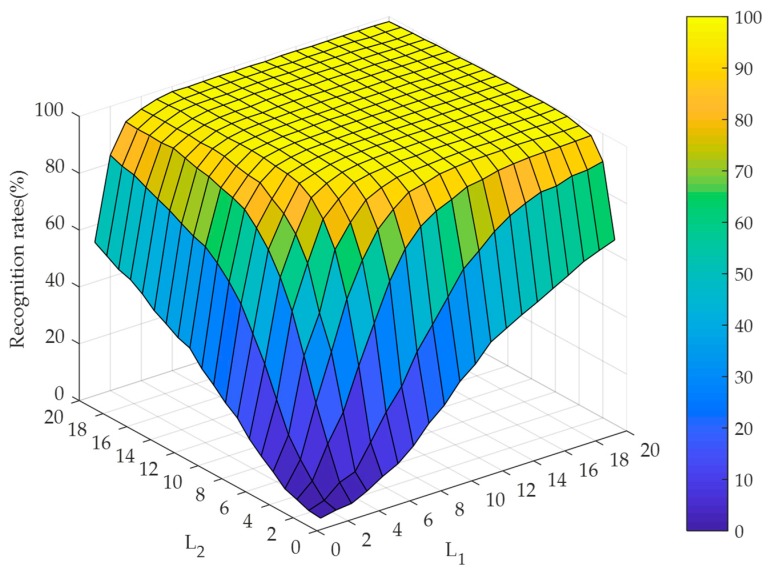
Recognition rates for RL2SR-TELM algorithm when the hidden node numbers of TELM vary from 1 to 20 in the case of noise-free.

**Figure 8:**
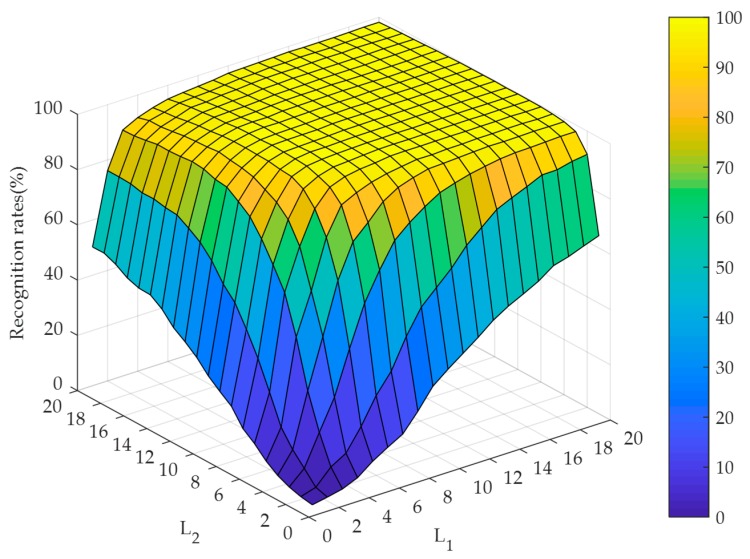
Recognition rates for RL2SR-TELM algorithm when the hidden node numbers of TELM vary from 1 to 20 in the case of white Gaussian noise contamination.

**Figure 9:**
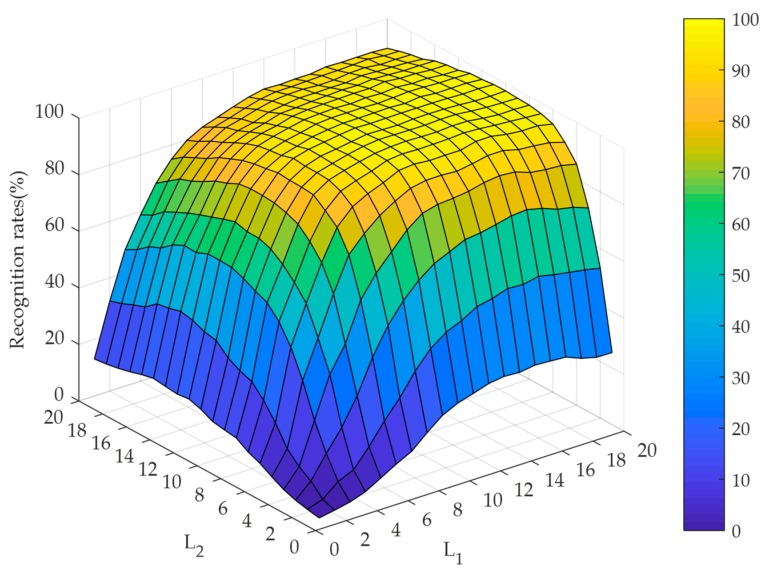
Recognition rates for RL2SR-TELM algorithm when the hidden node numbers of TELM vary from 1 to 20 in the case of 50% salt & pepper noise contamination.

To evaluate the effect of the hidden node numbers along the directions of TELM, the experiments are implemented by setting the hidden node numbers varying from 1 to 20 under the cases of noise-free and different noise contaminations. The recognition performance is illustrated in Figure 7, Figure 8 and Figure 9. At the same time, Figure 7 and Figure 8 illustrate that our algorithm could converge rapidly with the increase of hidden node numbers. Obviously, when the hidden node numbers are both greater than 7, our algorithm achieves a perfect performance. From Figure 9, although the convergence performance is inferior to the noise-free case, our algorithm can still obtain better convergence speed. As a consequence, the appropriate hidden node numbers can be selected according to the above analysis. For simplicity, the hidden node numbers in our experiments are set as $L_{1}=L_{2}=10$.

### 4.3. Experiment Results and Analysis

In this subsection, the experiments are implemented to validate the efficiency of our presented algorithm from the aspects of sparse representation, fusion strategy, classification approach and the overall algorithm. For the sake of demonstrating the robustness of the presented RL2SR model, we accomplish the experiments compared with several different models, such as SRC, CRC and DSRM. The recognition rates are shown in Table 2.

From Table 2, it is easy to discover that each algorithm achieves the highest and the lowest recognition rates under the cases of noise-free and salt & pepper noise contamination, respectively. Since our proposed adaptive weighted fusion process approximates a spatial smoothing filtering, the decrease of recognition rate under the white Gaussian noise contamination is not obvious. Furthermore, by using our RL2SR coefficient for fusion, the recognition rates achieve 99.68%, 99.20% and 97.24%, which are 1.72%, 2.52 and 2.96% higher than DSRM under the cases of noise free, white Gaussian noise and SRC in the case of salt & pepper noise contamination, respectively. This indicates that our RL2SR is robust to different noises, which can improve the discriminant competency and increase the recognition rate of the fusion image.

To evaluate the efficiency of the presented adaptive fusion strategy, some comparison fusion experiments (i.e., the sum and min-max fusion strategy) are simulated and the recognition performance is listed in Table 3. In this experiment, the training sample number of each class varies from 2 to 4.

Table 3 illustrates that the recognition accuracies under different fusion strategies increases with the training sample number. In particularly, our presented fusion strategy achieves the highest recognition accuracy of 100%, 99.95% and 99.05% when we set the number of training samples as 4. Even when the training sample number declines to 2, our approach achieves an accuracy of 92.27% which is 19.74% higher than the min-max fusion strategy in the case of salt & pepper noise contamination (72.53%). This implies that our fusion strategy has the strongest robustness compared with the sum and min-max fusion methods.

**Figure 10:**
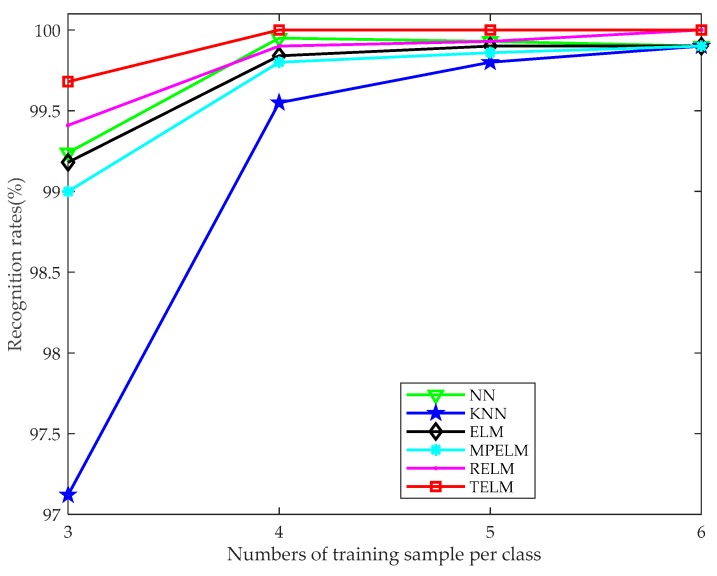
Recognition rate curves for different classifiers when the training sample number varies from 3 to 6 in the case of noise-free.

To demonstrate the classification efficiency of the presented TELM, we accomplish the experiments compared with some other classifiers, such as NN, KNN, ELM, MPELM and RELM. For these comparison classifiers, we vectorize the fused image and take this vector as the input. For each classifier, 3–6 training samples are selected to complete the recognition experiments and the classification accuracy curves are plotted in Figure 10.

The curves in Figure 10 indicate that when the training sample number is greater than or equal to 4, the recognition rates of all the algorithms achieve excellent performance. The experimental results also show that, in the case of noise-free, the recognition rate of our proposed TELM algorithm gradually increases with the number of training samples. On the other hand, our TELM achieves higher recognition rates than the other algorithms. Although the improvement is not significant because the recognition rate is much approximate or even reaches to 100%. From the above analysis, it is easy to observe that the presented TELM algorithm can achieve efficient recognition performance and has strong stability compared with the other classifiers. Furthermore, more simulation experiments are implemented with the multispectral palmprint database when it is contaminated by the aforementioned noise. The recognition performances are illustrated in Table 4.

It is observed from Table 4 that, in the case of white Gaussian noise contamination, the recognition rate of TELM outperforms the other classifiers. Meanwhile, the recognition accuracy of the presented TELM is remarkably higher than the other methods under the case of salt & pepper noise contamination. In consideration of the pulse characteristic of the salt & pepper noise, it impacts remarkably on the distance measurement between different samples. When the testing samples are contaminated by salt & pepper noise, the recognition accuracy of KNN method achieves 38.92%, which is significantly lower than our TELM algorithms (97.24%). Since the proposed TELM abandons the eigenvectors corresponding to the smaller eigenvalues which have the higher correlation to the noise contamination, and retains the principal components corresponding to the major eigenvalues, TELM has the ability of noise reduction and the better discrimination ability. The experimental results in Table 4 also validate that our algorithm can achieve the higher recognition rate and possess the stronger robustness to noise contamination compared with the other classifiers.

**Figure 11:**
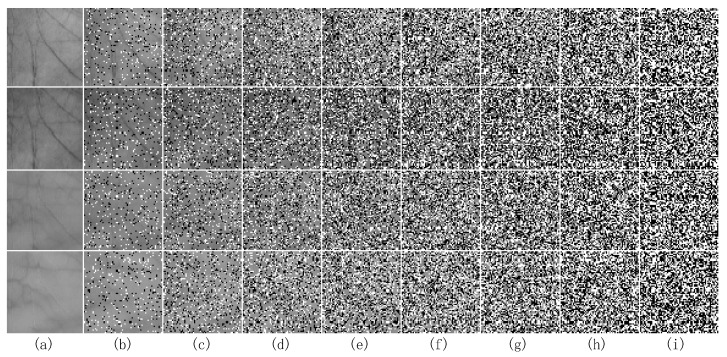
Some multispectral palmprint images contaminated by different percentages of salt & pepper noise. (**a**) is the original images under Blue, Green, Red and NIR spectrums. (**b**–**i**) are the images contaminated by 10–80% salt & pepper noise under different spectrums.

To further validate the robustness of the proposed TELM algorithm, we add different degrees of salt & pepper noise to the testing sample and implement the recognition experiment. Figure 11 shows some noisy multispectral palmprint images contaminated by salt & pepper noise with 10% to 80% percentages. Figure 11a is the original images under different spectra. Figure 11b–i are the noisy contaminated images under different spectra when the degree of salt & pepper noise varies from 10% to 80%.

**Figure 12:**
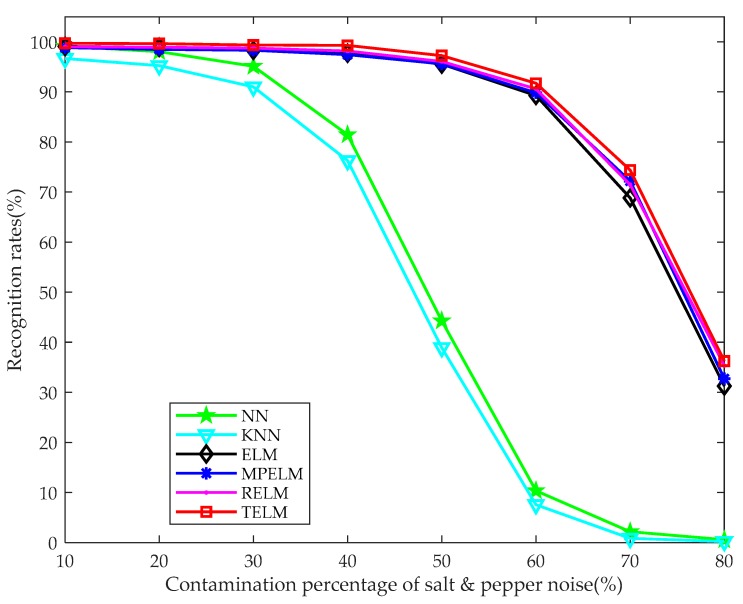
Recognition rates for different algorithms when the training sample number is 3 and the percentage of the salt & pepper noise contamination varies from 10% to 80%.

Figure 12 illustrates the recognition rate curves of our TELM algorithm and some of the aforementioned comparison classifiers. It is easy to find that the recognition rate curves of ELM MPELM, RELM and our algorithm drop significantly when the percentage of noise contamination is greater than 60%. Particularly, the recognition rate curves of NN and KNN methods are obviously lower than the other algorithms when the palmprint image is contaminated by more than 20% salt & pepper noise. That is to say, the accuracy curves of NN and KNN have the fast decline. The experiment result curves mean that our proposed TELM algorithm outperforms the comparison classifiers with different percentages of noise contamination and possesses stronger robustness.

Table 5 illustrates the average classification times of the aforementioned classifiers on the whole database. Although our TELM classifier is slower than the ELM method, the difference (i.e., 0.08 s) is very small. Moreover, it is distinctly faster than NN, KNN, MPELM and RELM classifiers. Especially, the classification time of NN is about five times that of our TELM. In additional, the above experiment results demonstrate that the recognition performance of our classifier significantly exceeds the NN, KNN, ELM, MPELM and RELM classifiers. This validates the recognition ability and efficiency of our algorithm.

Table 6 lists the recognition rates of our RL2SR-TELM algorithm with different spectral combinations. This experiment is implemented under the cases of noise-free, white Gaussian noise and 50% salt & pepper noise contamination and the training sample number per class is 4.

Table 6 summarizes the excellent performance of our presented algorithm in the cases of noise-free and white Gaussian noise contamination. In the noise-free case, the recognition accuracy achieves 100% for most of the spectral combinations. Even when the sample is contaminated by white Gaussian noise, our algorithm achieves the accuracy of more than 99.50% for all of the spectral combinations and 99.95% under the combination of Blue, Green, Red and NIR spectra. When the testing sample is contaminated by salt & pepper noise, the recognition rate declines significantly and achieves the lowest recognition rate 76.75% under the NIR spectrum. At the meantime, our RL2SR-TELM algorithm achieves an recognition performance under the combination of Blue, Green, Red and NIR spectra in the noise-free , white Gaussian noise and salt & pepper noise contamination cases, i.e., 100%, 99.95% and 99.05%, respectively. This indicates that our proposed RL2SR-TELM algorithm has excellent robustness to noise pollution.

Table 7 illustrates the recognition rates of our RL2SR-TELM algorithm compared with some state-of-the art palmprint recognition methods, such as deep scattering network method [18], texture feature-based method [42], and DCT-based features method [43] etc. It is easy to find that in the case of different training samples, our algorithm achieves an excellent recognition performance. Although the recognition accuracy of our algorithm is 0.32% lower than the deep scattering network method when the training sample number is three, and it is higher than the texture feature based method and DCT-based features method when the training sample number is four. Particularly, the recognition rate of our proposed algorithm reaches 100% when the number of training samples is greater than four.

Table 8 lists the recognition rates of our RL2SR-TELM algorithm comparing with some state-of-the-art multispectral palmprint recognition algorithms, such as matching score-level fusion by LOC method, DST-MPELM method, AE-RELM method, quaternion PCA using quaternion DWT method and image-level fusion by DWT method. In this experiment, we choose three samples per class to constitute the training set. The experimental results in Table 8 illustrate that our proposed algorithm can achieve an excellent recognition accuracy in the cases of both the noise-free (99.68%) and various noise contaminations (99.20% and 97.24%, respectively).

When the sample is contaminated by salt & pepper noise, the presented algorithm has more obvious advantages, which an accuracy that is respectively 0.76%, 7.26%, 1.48%, 7.08% and 14.49% higher than that of the other comparison algorithms. Table 9 demonstrates the time cost of our proposed RL2SR-TELM multispectral recognition algorithm for each test sample. It is easy to find that our RL2SR-TELM algorithm takes about 0.10945 s for a test sample recognition task.

**Figure 13:**
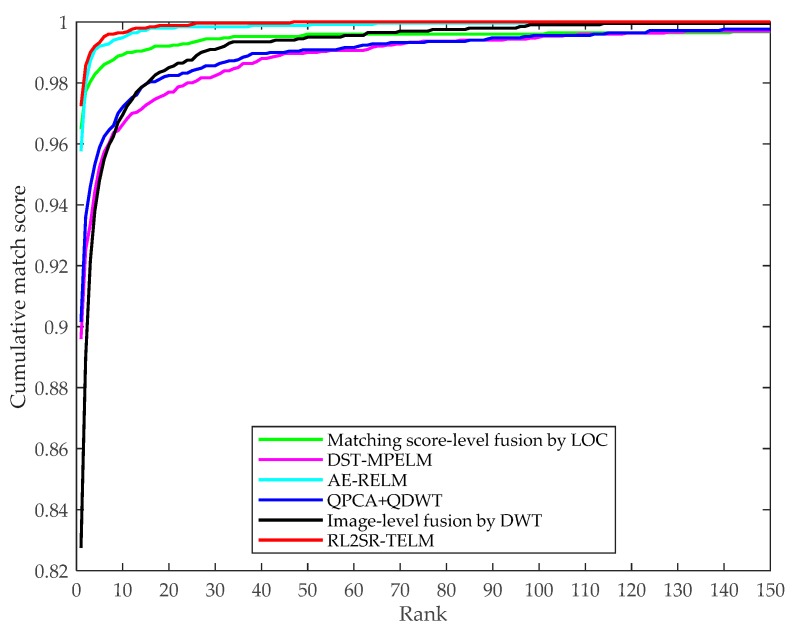
Performance for different multispectral palmprint recognition algorithms in terms of cumulative match characteristic curves.

To further demonstrate the performance of our presented RL2SR-TELM method, in the case of salt & pepper noise contamination, we plot the cumulative match characteristic (CMC) curves generated by our RL2SR-TELM method and the aforementioned comparison methods. Figure 13 shows the CMC curves.

From Figure 13, it is easy to find that our presented RL2SR-TELM method has the highest rank-1 recognition accuracy. Meanwhile, the cumulative match characteristic curve of our algorithm is mostly close to the upper left corner of the coordinate system comparing with the comparison multispectral palmprint recognition approaches which means that it has the rapidest convergence speed. This implies that our algorithm outperforms the others in recognition accuracy and noise robustness, and it is quite consistent with the aforementioned experiment results and analysis.

## 5. Conclusions

In this paper, a novel RL2SR-TELM algorithm is presented to implement multispectral palmprint recognition. Since the L2 regularization term is employed, the regularization optimal objective function is convex and a closed solution can be efficiently obtained. In addition, a new measurement, namely WSCCI, and an adaptive fusion framework are proposed to construct the fused multispectral palmprint images. For the classification task, we extend the conventional extreme leaning machine to the tensor domain and present a TELM algorithm. It deals with the palmprint image in two-dimensional space directly and makes the best use of its spatial structure to enhance the classification ability. Extensive experiments on PolyU multispectral palmprint database confirm the strong robustness, excellent recognition accuracy and high efficiency of our proposed algorithm.

## Figures and Tables

**Table 1:** Robust L2 sparse representation algorithm.

Input: testing sample $y^{l},(l=1,2,\ldots ,s)$, training sample matrix $X=[X_{1},\ldots ,X_{i},\ldots ,X_{C}]$, initiate the residual function matrix $W^{l,1}=diag(1,1,\ldots ,1)$. Output: linear representation coefficient $A^{l}$,(l=1,2,…,s).
While error not convergent, do 1. Calculate the collaborative representation code $\gamma^{l}$ by solving $$\gamma^{l,t+1}=\arg\underset{\gamma^{l}}{\min}{\left\|W^{l,t}(y^{l}-X\gamma^{l})\right\|}_{2}^{2}+\xi {\left\|\gamma^{l}\right\|}_{2}^{2}.$$ 2. Calculate the residual by employing $$e_{k}^{l,t+1}=\left|y_{k}^{l}-X_{k}\gamma^{l,t+1}\right|,(k=1,\ldots ,d).$$ 3. Calculate the residual function by using $$\omega (e_{k}^{l,t+1})=\frac{\exp(-\mu {\left(e_{k}^{l,t+1}\right)}^{2}+\mu \delta )}{1+\exp(-\mu {\left(e_{k}^{l,t+1}\right)}^{2}+\mu \delta )},(k=1,\ldots ,d).$$ 4. Update $W^{l}$ by utilizing $$W^{l,t+1}=diag(\omega (e_{1}^{l,t+1}),\omega (e_{2}^{l,t+1}),\ldots ,\omega (e_{d}^{l,t+1})).$$ 5. Calculate $error={\left\|W^{l,t+1}-W^{l,t}\right\|}_{F}/{\left\|W^{l,t}\right\|}_{F}.$ End while 6. For each spectral testing sample $y^{l},(l=1,2,\ldots ,s)$, calculate $A^{l},(l=1,2,\ldots ,s)$ by using $$A^{l}={\left(2\lambda CM+2\lambda X^{T}X+X^{T}W^{l}X\right)}^{-1}X^{T}W^{l}y^{l}.$$

**Table 2:** Recognition rates for different representation methods.

Representation Method	Recognition Rate (%)		
	Noise-Free	White Gaussian Noise	Salt & Pepper Noise
SRC	99.64	97.84	94.28
CRC	99.44	98.76	96.68
DSRM	97.96	96.68	96.28
RL2SR	99.68	99.20	97.24

**Table 3:** Recognition rates for different fusion methods when the training sample number per class varies from 2 to 4 under the cases of noise-free and different noise contaminations.

Fusion Method	Noise Contamination Case	Recognition Rate (%)		
		2	3	4
Sum fusion	Noise-free	97.50	99.56	99.90
	White Gaussian noise	96.70	99.44	99.65
	Salt & pepper noise	89.63	96.56	98.55
Min-max fusion	Noise-free	92.83	97.68	99.25
	White Gaussian noise	92.67	97.44	99.20
	Salt & pepper noise	72.53	82.16	85.85
Our adaptive fusion	Noise-free	97.73	99.68	100.00
	White Gaussian noise	97.47	99.20	99.95
	Salt & pepper noise	92.27	97.24	99.05

**Table 4:** Recognition rates for different classifiers under the cases of noise-free and noise contaminations.

Classifiers	Recognition Rate (%)		
	Noise-Free	White Gaussian Noise	Salt & Pepper Noise
NN	99.24	96.48	44.24
KNN	97.12	93.32	38.92
ELM	99.18	99.16	95.55
MPELM	99.00	98.80	95.60
RELM	99.41	98.96	96.07
TELM	99.68	99.20	97.24

**Table 5:** Classification time for different classifiers.

Classifiers	Classify Time (s)
NN	7.76
KNN	5.17
ELM	1.51
MPELM	1.82
RELM	1.67
TELM	1.59

**Table 6:** Recognition rates for our RL2SR-TELM with different spectral combinations under the cases of noise-free and different noise contaminations.

Spectral Combination	Recognition Rate (%)		
	Noise-Free	White Gaussian Noise	Salt & Pepper Noise
Blue	99.55	98.65	80.90
Green	99.50	99.25	87.65
Red	99.45	99.15	83.10
NIR	98.65	94.50	76.75
Blue, Green	100.00	99.80	95.80
Blue, Red	99.95	99.80	93.30
Blue, NIR	100.00	99.85	90.70
Green, Red	99.75	99.50	96.15
Green, NIR	100.00	99.80	95.80
Red, NIR	99.90	99.90	96.60
Blue, Green, Red	100.00	99.85	98.65
Blue, Green, NIR	100.00	99.90	97.60
Blue, Red, NIR	100.00	99.90	97.15
Green, Red, NIR	99.95	99.85	98.35
Blue, Green, Red, NIR	100.00	99.95	99.05

**Table 7:** Recognition rates for different multispectral palmprint recognition algorithms in the case of noise-free.

Algorithm	Recognition Rate (%) for Different Training Sample Number			
	3	4	5	6
Deep scattering network method [18]	100	100	100	100
Texture feature based method [42]	-	99.96	99.99	100
DCT-based features method [43]	-	99.97	100	100
Our proposed RL2SR-TELM	99.68	100	100	100

**Table 8:** Recognition rates for our RL2SR-TELM and some other multispectral palmprint recognition algorithms.

Algorithm	Recognition Rate (%)		
	Noise-Free	White Gaussian Noise	Salt & Pepper Noise
Matching score-level fusion by LOC [41]	99.43	99.23	96.48
DST-MPELM [39]	99.47	98.30	89.98
AE-RELM [38]	99.16	98.48	95.76
QPCA + QDWT [37]	98.83	93.33	90.16
Image-level fusion by DWT [32]	99.03	96.23	82.75
Our proposed RL2SR-TELM	99.68	99.20	97.24

**Table 9:** Time cost of our RL2SR-TELM algorithm.

Procedure	RL2SR and Adaptive Fusion	TELM	Total Time
Average time (s)	0.10892	0.00053	0.10945
